# A Randomized, Placebo-Controlled Clinical Trial of Famciclovir in Shelter Cats with Naturally Occurring Upper Respiratory Tract Disease

**DOI:** 10.3390/ani10091448

**Published:** 2020-08-19

**Authors:** Chelsea L. Reinhard, Emily McCobb, Darko Stefanovski, Claire R. Sharp

**Affiliations:** 1 Department of Clinical Sciences and Advanced Medicine, University of Pennsylvania School of Veterinary Medicine, Philadelphia, PA 19104, USA; 2 Department of Clinical Sciences, Cummings School of Veterinary Medicine at Tufts University, North Grafton, MA 01536, USA; emily.mccobb@tufts.edu; 3 Department of Clinical Studies, New Bolton Center, University of Pennsylvania School of Veterinary Medicine, Kennett Square, PA 19348, USA; sdarko@vet.upenn.edu; 4 School of Veterinary Medicine, College of Science, Health, Engineering and Education, Murdoch University, Murdoch 6150, Australia; c.sharp@murdoch.edu.au

**Keywords:** feline upper respiratory infection, cat, animal shelter, feline herpesvirus, shelter medicine, antiviral

## Abstract

**Simple Summary:**

Upper respiratory tract disease (URTD) impacts the health and welfare of shelter cats. Mitigation strategies include stress reduction and population-level approaches; effective treatment plans focus on reducing the clinical signs in cats affected by URTD. This study evaluated the use of famciclovir, an antiviral therapy, in reducing clinical signs in shelter cats with URTD when administered at a target dose range of 40–90 mg/kg twice daily for up to 21 days. Cats were randomized into either a famciclovir treatment group (*n* = 11) or placebo group (*n* = 11). Testing for viral pathogen identification was performed at enrollment in the study, and clinical scoring was completed daily to evaluate the severity of signs. With each day of treatment, cats in both groups were less likely to experience worsening clinical scores; however, cats in the famciclovir treatment group had a significantly lower risk of worsening clinical signs with each day compared to the placebo group. Feline herpesvirus, a common pathogen causing URTD, was identified in 11/21 cats. The results of this small study justify the need for further research to determine the utility of famciclovir as part of treatment protocols for improving clinical signs and overall impacts of URTD in shelter cats.

**Abstract:**

Upper respiratory tract disease (URTD) is a clinically relevant infectious disease in shelter cats, with individual and population-level welfare implications. The purpose of this study was to evaluate the effectiveness of famciclovir in reducing clinical signs of URTD in shelter cats during a therapeutic period of up to 21 days. Cats at two Northeastern United States animal shelters with URTD clinical signs were enrolled in a pragmatic, prospective, randomized, placebo-controlled clinical trial. Cats received either famciclovir (*n* = 11, target dose range 40–90 mg/kg) or placebo (*n* = 11), administered orally twice daily for up to 21 days with once-daily clinical scoring. At enrollment, conjunctival and oropharyngeal samples were collected for respiratory pathogen identification by RT-PCR. Zero-inflated Poisson regression was used to evaluate the treatment group effects and changes in clinical scoring over time. With each day of treatment, cats in both groups were less likely to experience worsening clinical scores; however, the risk of worsening scores with each day of treatment was significantly less in the famciclovir group compared to placebo (*p* = 0.006). Feline herpesvirus (FHV-1) DNA was detected in 11/21 cats. The findings justify further pragmatic studies to determine whether famciclovir treatment can contribute to a clinically relevant reduction in URTD morbidity in shelter cats.

## 1. Introduction

Upper respiratory tract disease (URTD) has been suggested as a leading cause of feline euthanasia in animal shelters [1] and is recognized as one of the main disease concerns among shelter staff [2]. Feline herpesvirus (FHV-1) and feline calicivirus are the most common pathogens found to cause URTD [3,4]. In exposed cats, FHV-1 has a reported incubation period of 2 to 6 days [5]. Recrudescence due to stress in cats with latent infections [6] further complicates FHV-1-induced disease in shelters, as studies have associated stress with URTD in cats [7,8]. One study found substantial increases in FHV-1 shedding one week after intake in shelter cats [9]. Increased URTD morbidity in shelter cat populations, characterized by clinical signs including nasal and ocular discharge and sneezing, leads to welfare concerns, resource allocation issues, and increasing length of stay.

Population-level management strategies can reduce stress, such as minimizing primary enclosure movement within the facility, which has been associated with a lower risk of URTD [10]. One study highlighted the importance of housing features that promote welfare in reducing stress levels among shelter cats [11]. Another study showed that shelter cats receiving human interactions had a lower incidence of URTD compared to a control group [12]. Other mitigation strategies address known risk factors for URTD in feline populations, including inadequate sanitation practices and overcrowding [13]. Eliminating URTD morbidity completely can be challenging, and utilizing medical treatment to minimize the severity and hasten resolution of clinical signs is important to improving individual animal health and welfare, as well as having population-level impacts. Population monitoring and prompt identification of cats with URTD are critical in the shelter environment to promote population health [14,15]. Supportive care and antimicrobial therapy are common in URTD treatment plans, and one study of North American shelters found that doxycycline and amoxicillin/clavulanic acid were the most frequently chosen antimicrobials for the treatment of URTD in shelter cats [16].

Antiviral therapy has been considered as a promising treatment option for URTD in cats, but to date, clinical evaluation of its use in cats has been limited. The antiviral agent famciclovir and its active metabolite penciclovir have been studied as a treatment for FHV-1 in vitro, in experimental FHV-1 infection, and in naturally occurring URTD in both client-owned and shelter cats. Numerous in vitro studies of penciclovir have documented antiviral efficacy against FHV-1 [17,18,19,20]. In vivo, famciclovir has complex and non-linear pharmacokinetics [21,22,23]. In a pilot study, a famciclovir dose of ~40 mg/kg three times daily resulted in variable tear famciclovir concentrations in cats [24], while in another study, a dose of 90 mg/kg q12h achieved plasma and tear concentrations likely to be therapeutic for FHV-1 treatment [23].

Famciclovir has been shown to be effective in treating experimentally FHV-1-infected cats, resulting in lower median clinical disease scores compared to cats receiving placebo [25]. Additionally, the efficacy of oral famciclovir has been reported in two retrospective case series of client-owned cats with suspected or confirmed FHV-1 infection, with minimal adverse effects [26,27]. In contrast to the aforementioned studies, in a recent randomized study of shelter cats with URTD, administration of famciclovir at ~30 mg/kg or ~90 mg/kg q12h for seven days did not result in improved clinical scores compared to placebo or no treatment but was associated with lower FHV-1 conjunctival shedding on days eight and 15 [28]. Prophylactic oral administration of famciclovir to shelter cats has not been shown to reduce the incidence of URTD [28,29]. Given the current state of clinical equipoise, further studies are needed to clarify the role of famciclovir in the treatment of clinical URTD in shelter cats.

The objective of this prospective multi-shelter clinical trial was to evaluate the effectiveness of famciclovir in reducing observed clinical signs of URTD in shelter cats during a therapeutic period of up to 21 days. We hypothesized that famciclovir administered at a dose within the range of 40–90 mg/kg using commercially available products orally twice daily for up to 21 days, compared to placebo, would reduce the duration and severity of URTD clinical signs, based on a clinical scoring system, in shelter cats.

## 2. Materials and Methods

### 2.1. Study Sites

The study was approved by the Institutional Animal Care and Use Committee of Tufts University (protocol #G2015-126). Cats were enrolled, and data collection was conducted at two animal shelters in the Northeast United States during the study period from May 2016 to September 2017. Inclusion criteria included clinical signs consistent with URTD, as documented by a shelter veterinarian, including one investigator, at time of enrollment, and the bodyweight of 1.5 kg or greater. Clinical signs consistent with URTD included nasal discharge, ocular discharge, sneezing, open mouth breathing, and oral ulcerations. Administration of study medication, clinical scoring, and collection of upper respiratory tract samples were performed by trained shelter veterinarians or shelter staff. The on-site shelter veterinarians and shelter staff were trained didactically in the use of the clinical scoring system for the interpretation of clinical signs. Due to the pragmatic design of the study, the same individual was not available for each day of treatment and clinical scoring. In a two-sided sample size calculation, it was determined that 16 cats would be needed in each treatment group to identify a reduction in the duration of clinical signs from nine days to seven days (assuming a standard deviation of two days), with a power of 0.8 and an alpha of 0.05. The nine-day average duration of URTD clinical signs in shelter cats was based on the findings from a previous observational study of 100 cats performed by one of the authors (unpublished). We aimed to enroll 25 cats per group to account for dropout.

### 2.2. Clinical Trial and Data Collection

Using separate randomization plans generated by an online program (http://www.randomization.com) for each shelter, cats were assigned to either the famciclovir or placebo group. One investigator created study packets that included data collection sheets for each sequentially enrolled cat and the treatment allocation (designated A or B). Upon enrollment, the staff at each shelter used the next sequentially numbered study packet, informing them of the blinded treatment allocation for that cat. Commercially available famciclovir tablets (Apotex Corp., Weston, FL, USA, and Teva Pharmaceuticals USA, North Wales, PA, USA) and placebo tablets (lactose with sucrose for binding; Homeopathic Laboratories, King of Prussia, PA, USA) were administered according to a dosing chart (Table 1) twice daily by mouth, such that the resultant famciclovir dose would range from ~42 to 83 mg/kg. The medication was administered for the duration of enrollment, which was designated as 21 days or until the cat could no longer be in the study due to pathway (e.g., adoption, foster, etc.), whichever occurred sooner. Treatments were not identical in tablet size, but the bottles were labeled to mask identification and administered using various methods: by hand, commercially available devices for pill administration, and/or hidden in commercially available chews or canned cat food. Treatments were scheduled on a treatment sheet for each cat, and sheet review was performed by study investigators at the completion of the study to assess protocol compliance. If treatment was not checked off on the sheet, it was considered that the treatment was not administered.

A clinical score was assigned to each cat at the time of enrollment, and the scoring was continued once daily for the duration of enrollment. Daily clinical scoring was performed at the time of medication by the same shelter veterinarian or staff member who was administering the treatment. The clinical scoring system was adapted by one of the authors from a published scoring system [30] (Table 2). The total daily clinical score was a cumulative score of all categories (ocular discharge, nasal discharge, sneezing, oral ulceration, ulcer(s) on lips/nares, dyspnea with audible rales, open mouth breathing, and dehydration), where a score of zero represented no clinical signs, and a score of 29 was the most severe clinical signs.

The previously published clinical scoring system on which ours was based was reported in a study investigating the efficacy of a trivalent feline vaccine against FHV-1, feline calicivirus, and feline panleukopenia [30]. Additionally, those authors reported that the scoring system had been approved by the Center for Veterinary Biologicals in the original immunogenicity investigations for the licensure of the vaccine [30]. This scoring system was adapted to remove some redundancy and aid in usefulness for true daily scoring since the previous score assigned weight to the number of days a given clinical sign had been present such that daily scores were not independent observations. In addition to removing the weighting for the duration of clinical signs, we removed some score components: salivation, as it is likely closely linked to ulcer severity, hypothermia, as body temperature is not taken daily in shelter cats, and coughing, as this is likely to be an intermittent clinical sign that could be missed during a short observation period. We also removed the reference to conjunctivitis but retained ocular discharge in the score, and for both the ocular and nasal discharge, retained greater weighting in the score for mucopurulent versus serous discharge. We also retained weighting of oral ulcer severity based on size and number, and lip/nose ulcer severity based on the presence or absence of bleeding.

At the time of clinical scoring, observations were also made for adverse effects that could potentially be attributed to the study medication, including vomiting, diarrhea, and decreased appetite. Concurrent treatments, including antibiotics, were recorded, as were the approximate age and sex of enrolled cats. Cats were housed and fed according to each shelter’s respective protocols. Much of the feline housing at the shelter locations included single-occupant enclosures, some double-sided. Shelter veterinarians and staff could withdraw a cat from the study due to potential adverse effects or inability to medicate the cat.

At the time of enrollment, conjunctival and oropharyngeal samples were collected using sterile cotton swabs with plastic ends from each cat. The two swabs were placed together in sterile red top tubes and stored at 4 °C before transport on ice to a commercial laboratory for a feline upper respiratory disease RT-PCR panel (IDEXX Laboratories, Westbrook, ME, USA, test code 2512).

### 2.3. Statistical Analysis

Continuous variables are reported using the median (range) unless otherwise specified. Frequency counts and percentages are used for categorical variables (e.g., sex, signalment). Tests of normal distribution (Shapiro-Wilk normality test) were performed to determine the extent of skewness, and transformation methods (e.g., logarithmic) were used to normalize the distribution of seriously skewed variables. The a priori plan for statistical analysis of the primary outcome measure of the duration of clinical signs was to compare group means using an independent group t-test for normally distributed continuous data, or medians with a Mann-Whitney U test if non-parametric. However, during data collection, it became apparent that a proportion of cats had URTD clinical signs extending to the end of the study period, and others had signs that would resolve (score 0), before returning, such that there was a large proportion of zero scores. As such, an alternative statistical methodology was sought to address both the duration and severity of clinical signs and account for the zero scores.

Data were analyzed on an intention-to-treat basis. Zero-inflated Poisson (ZIP) regression was considered the most appropriate methodology to evaluate the treatment group effects and changes to clinical scoring over time. The URTD clinical scoring system used satisfied two characteristics that made it appropriate for analysis with Poisson regression and a subsequent third characteristic that made the ZIP approach appropriate. First, the score was only receiving integer values that were greater than or equal to zero; such data were considered count data. Second, based on visual inspection of a histogram of the data, it was established that the score exhibited exponential distribution (i.e., non-normal distribution). Log-transform to achieve normality was not considered appropriate as all the zero values would be lost. Finally, a ZIP model was considered appropriate since a substantial portion of the clinical score data was zero [31]. As part of the multivariable ZIP model, the time when the score was assessed as a continuous independent variable was considered a fixed-effects confounder. Statistical interaction between the treatment and time in days was also included in the model. Other explanatory variables included were treatment, estimated age in years, sex, and shelter location. The incidence rate ratio (IRR) was calculated as the exponential of the respective coefficients associated with specific independent variables in the ZIP multivariable model. Post hoc analysis was used to determine the model adjusted effects and marginal means. The analysis was conducted with Stata 16MP (StataCorp, College Station, TX, USA) with two-sided tests of hypotheses and a *p*-value < 0.05 as the criterion for statistical significance. The biostatistician (author D.S.) was blinded to the treatment groups for the analysis work.

## 3. Results

### 3.1. Descriptive Data

A total of 22 cats were enrolled and included in the analysis (famciclovir group: *n* = 11, placebo group: *n* = 11) at two shelters (designated A and B). One additional cat (randomized to the placebo group) from shelter B enrolled without daily clinical scoring was excluded from the analysis. Due to an unexpectedly low prevalence of the disease of interest among the study population, we were unable to enroll the target number of cats within a reasonable study time frame and proceeded with this sample size for analysis. Demographic data pertaining to cat sex, shelter location, and estimated age, in addition to antibiotic administration during enrollment in the study, are displayed in Table 3. Utilizing the famciclovir dosing chart, the median famciclovir dose administered to 10 of 11 cats was 57 mg/kg (range = 45–66 mg/kg). Weight was not available for one cat on record review, and thus the mg/kg dose received by that cat is unknown.

All 22 cats were included in the intention-to-treat analysis. Major protocol violations were reported for three cats in the famciclovir group. Two cats were withdrawn from the study from shelter A; one cat due to inability to administer any treatments, and the other due to hypersalivation and vomiting after being administered famciclovir. The third cat, from shelter B, was randomized to receive famciclovir, however received a placebo for the duration of enrollment.

Minor protocol violations were also reported as missed treatments during the time spent in the study. Over half of the cats had at least one treatment missing as indicated on the available treatment documentation, with higher frequency and number of missed treatments observed for the famciclovir group (famciclovir group: *n* = 6; placebo group: *n* = 5). Two cats in the famciclovir group and one cat in the placebo group did not have a treatment sheet available for review after the study. The median percentage of missed treatments for the famciclovir group was 9.0% (range = 2.4–100%), with the cat withdrawn due to the inability to medicate, representing the maximum of the range. This was compared to a median percentage of 2.4% (range = 2.4–11.1%) for the placebo group. One cat was removed from the study after nine days due to temperament but was not considered to be a protocol violation due to variable lengths of study enrollment observed and data collection during the time in the study.

### 3.2. Clinical Scoring and Duration of Enrollment

**Figure 1:**
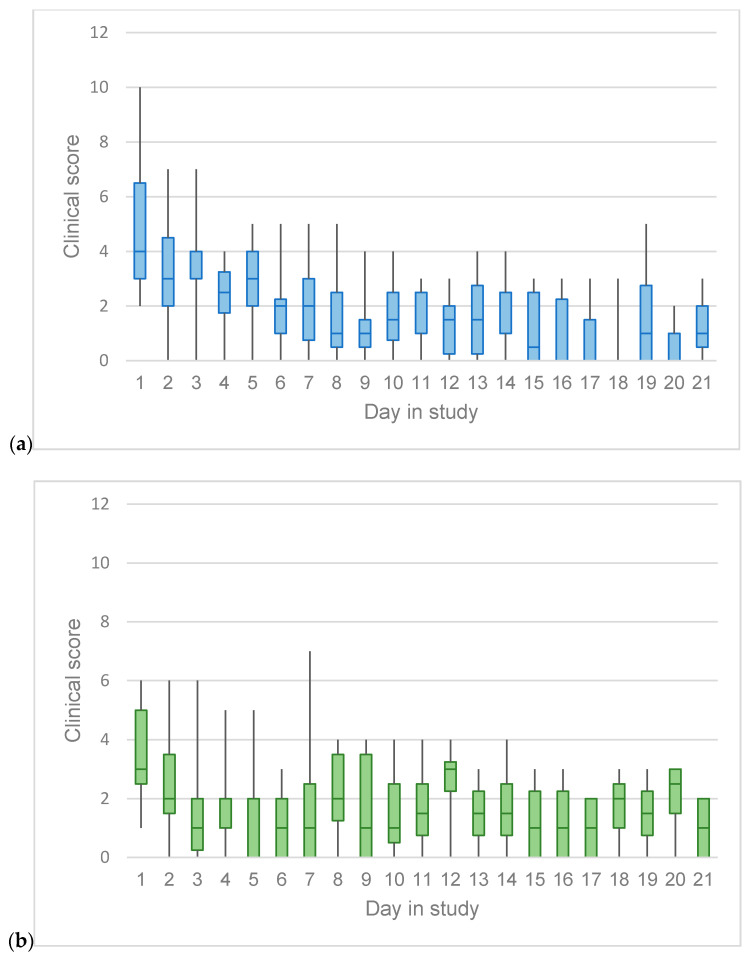
Median clinical scores (middle line), interquartile ranges (boxes), and minimum and maximum (whiskers) clinical scores of shelter cats with upper respiratory tract disease randomized to receive famciclovir (**a**) or placebo (**b**). Eleven cats were represented on day one in both the famciclovir and placebo groups (intention-to-treat), with cat numbers by day varying on the number of days in the study.

Clinical signs of URTD were considered mild-to-moderate in the cats enrolled such that at no point during the study did any cat have a clinical score greater than 10. Cats in the famciclovir group had a higher median clinical score at the time of enrollment in the study (Figure 1a) than the placebo group (Figure 1b). With each day of treatment, cats in both groups were less likely to experience worsening observed URTD clinical signs. Over time, cats in the famciclovir group were less likely to experience higher scores (IRR: 0.918 per day; *p* < 0.001; 95% CI: 0.896–0.940). In a similar fashion, cats in the placebo group were less likely to experience higher scores over time (IRR: 0.962 per day; *p* = 0.002; 95% CI: 0.937–0.986). In post hoc pairwise comparison, the risk of worsening clinical signs per day of treatment was significantly less in the famciclovir group compared to the placebo group (*p* = 0.006). The effect of excluding cats with major protocol violations was not found to be significant (*p* = 0.168) and did not significantly change the IRR for either group.

Cats in the famciclovir group had a longer median duration of study enrollment (Table 3). Seven cats in total reached the end of the 21-day study, at which time 2/3 cats in the famciclovir group (clinical scores of 1 and 3) and 2/4 cats in the placebo group (both with clinical scores of 2) still had clinical signs of URTD.

RT-PCR was performed on 21 of the 22 cats at the time of enrollment; one cat’s samples were not submitted when the cat was withdrawn from the study. Of the 21 cats, RT-PCR detected FHV-1 DNA in 11 cats—five in the famciclovir group and six in the placebo group. The majority of the FHV-1-positive cats had less than 38,000 viral particles detected, suggestive of a latent infection based on laboratory interpretation (IDEXX Laboratories). Feline calicivirus, *Chlamydophila felis*, *Bordetella bronchiseptica*, and *Mycoplasma felis* were detected in cats in both groups (Table 4).

## 4. Discussion

In this small study, using ZIP regression, we documented that while cats in both famciclovir and placebo groups experienced a decreased risk of worsening clinical signs over time, the risk of worsening clinical signs with each day of treatment was significantly less in the famciclovir group compared to the placebo group in cats treated orally for up to 21 days. Only one cat experienced hypersalivation and vomiting after famciclovir dosing, consistent with the previously reported favorable safety profile of the drug in cats.

These results, reported as an intention-to-treat analysis, are particularly remarkable, given the high number of protocol violations. While protocol violations are not ideal, the persistence of a treatment effect despite them suggests the potential for a clinically relevant outcome. Given the pragmatic design of this study, the observed protocol violations may reflect challenges in the shelter setting. It also could highlight the difficulty in administering famciclovir to cats in this study or aversiveness to medicating, supporting considerate patient selection. This study demonstrates that famciclovir has the potential to benefit shelter cats with URTD, even if some doses are inadvertently missed.

A famciclovir treatment effect was also evident despite the famciclovir dose being lower than intended. The target dose range was 40–90 mg/kg twice daily, but the median dose achieved was only 57 mg/kg (range = 45–66 mg/kg). This reflects a limitation in our original dosing chart but nonetheless suggests that pragmatic dosing of a halved tablet (125 mg total) of famciclovir for small cats (<3 kg) and one tablet (250 mg) of famciclovir for adult cats (up to 6 kg) may be adequate to result in a clinical benefit. While there is not yet data regarding whether the underdosing of famciclovir could lead to the development of famciclovir resistance in FHV-1, underdosing of antibiotics has been shown to promote resistance among bacteria, and consideration should be taken for this concern in antiviral therapies [32]. Further studies that address the potential for antiviral resistance could ultimately inform decisions regarding the dosing of famciclovir in shelter cats.

The treatment effect was evident despite two shelter locations with different supportive care protocols and varied administration of antibiotics. When considering the population of cats with URTD reported herein, the low clinical scores observed, the relatively low prevalence of FHV-1 by RT-PCR, and the low viral shedding suggestive of recrudescence of latent infection may have all resulted in an underestimation of the broader treatment effect of famciclovir. It is possible that in cat populations with more severe clinical signs, higher prevalence of FHV-1, and a greater proportion of new FHV-1 infections, treatment with famciclovir may have greater clinical benefit. On the contrary, it is possible that in populations with lower FHV-1 positivity, famciclovir may have no treatment benefit. This study was based on the empirical treatment approach with famciclovir, which we believe will be informative for shelters. Evidence-based decision-making informs the empirical therapy choices often made for URTD in shelter cats, as RT-PCR testing is not routinely performed for all individual shelter cats with URTD.

One difference between our study and a previous randomized study of famciclovir in shelter cats is the duration of treatment [28]. Our study continued treatment for up to 21 days, compared with seven days of treatment in the previous study, where a difference in clinical score was not observed between groups [28]. We planned to continue therapy for up to 21 days based on a previous experimental study of FHV-1 infection [25]. However, given our goal to prevent shelter pathway impediments for cats in the study, the duration of enrollment of individual cats was varied, achieving a median of 18 days treatment in the famciclovir group. The lack of standard duration of enrollment in the study could have resulted in an underestimation of the famciclovir treatment effect. Additionally, a longer duration of famciclovir administration may be indicated in some cats, given that seven cats in this study still had URTD clinical signs at 21 days. Nonetheless, the approach to the duration of therapy adopted in this study, for up to 21 days or until appropriate based on the shelter pathway, is likely practical for shelters to implement. We were unable to directly assess the impact of the treatment on length of stay with this study design, but improvement in clinical signs has the potential to reduce the length of stay for shelter cats with URTD. Since the length of stay is an important outcome measure in shelter cats, it should be investigated in future studies of famciclovir treatment.

This study had numerous additional limitations. Firstly, the nature of the clinical score data was more complex than anticipated a priori since some cats failed to achieve resolution of their URTD clinical signs during the study period, and some had clinical signs that waxed and waned, rather than consistently improving. This resulted in the use of a statistical methodology that best represented the obtained data but precluded us from definitively answering the question of whether or not famciclovir should be added to the empirical treatment protocols of shelter cats with URTD. Nonetheless, our findings do support the ongoing investigation of the utility of famciclovir in this setting. Secondly, although blinded treatments were intended, the treatments were not identical in tablet size, and it is possible that some shelter staff were familiar with the commercially available famciclovir products used in the treatment group, impacting the interpretation of the clinical scoring. Another limitation is that a ‘no treatment’ group was not included, as was done in a previous study [28]. The rationale for a ‘no treatment’ group is that the administration of oral medications to cats can be stressful and may exacerbate URTD; thus, it is possible that a ‘no treatment’ group might have experienced an improved clinical course relative to the famciclovir and placebo groups in our study. Additionally, the clinical scores in this study were reported as a total and did not allow us to further investigate specific (e.g., nasal vs. ocular) clinical signs exhibited by the cats. This also limited our ability to evaluate whether there were differences in response to treatment based on specific clinical signs exhibited, which could be addressed in future research by tracking category scores in addition to cumulative scores. Although the shelter staff underwent training in the use of the clinical scoring system with one of the investigators, we did not attempt to measure the inter-observer reliability of the score in this study. Because there were multiple shelter staff members contributing to the data collection and two shelter sites, there could be variation in reported scores based on subjectivity in the scoring system.

Cats in the placebo group of this study did show improvement in clinical scores over time. The small potential benefit of empirically adding famciclovir to the treatment protocols for shelter cats with URTD that is suggested by this study needs to be weighed against the cost, staff training and time, severity of illness, potential for antiviral resistance, risk of gastrointestinal upset, and possible stress of medicating, especially as the role of stress in URTD development is well documented. It is important to consider the clinical implications of the study results we present and whether the use of famciclovir in the treatment of shelter cats with URTD may have benefits in a given population. Numerous features of this clinical trial also limit its generalizability, including the study location in the northeast of the United States, the involvement of only two shelters, a small sample size, and a low number of cats eligible for enrollment. The number of cats available for enrollment over the study period was lower than anticipated, based on the previously reported incidence of URTD in shelter cats [1]. This could have been due to population-based management strategies being utilized in these shelter settings and/or other features of these populations.

The ongoing evaluation of famciclovir, in addition to standard URTD treatment, in further pragmatic studies in shelter cat populations, would aid in determining its utility in shelter environments. In future studies, it would be beneficial to employ a larger sample size across more shelters, focus on a more clinically affected population, include a ‘no treatment’ group, and analyze length of stay as an outcome measure.

## 5. Conclusions

In the present study of shelter cats with URTD, the administration of famciclovir at a median dose of 57 mg/kg (twice daily for up to 21 days) resulted in a lower risk of worsening clinical signs with each day of treatment compared to placebo. Our findings justify further pragmatic studies to determine whether famciclovir treatment can contribute to a clinically relevant reduction in URTD morbidity in shelter cats.

## Figures and Tables

**Table 1:** Cat body weight at the time of study enrollment was utilized to determine target dosing of famciclovir between 40 and 90 mg/kg, designed for tablet administration using commercially available products in a shelter setting.

Weight (kg)	Dose of 250 mg Tablet Famciclovir (Orally Twice Daily)	Famciclovir Dose (mg/kg) for Weights in Range
1.5–3.0 kg	½ tablet	83.33–41.67 mg/kg
3.01–6.0 kg	1 tablet	83.06–41.67 mg/kg
6.01–9.0 kg	1 ½ tablets	62.40–41.67 mg/kg

**Table 2:** Clinical scoring system for feline upper respiratory tract disease adapted from the published scoring system [30].

Category	Clinical Sign	Absent	Daily Score		
			Mild	Moderate	Severe
1. Ocular discharge	Serous	0	1	2	3
	Mucopurulent	0	2	3	4
2. Nasal discharge	Serous	0	1	2	3
	Mucopurulent	0	2	3	4
3. Sneezing		0	1	2	3
4. Oral ulceration	Single ulcer < 4 mm	0	2		
	Multiple ulcers < 4 mm	0	3		
	Any ulcer ≥ 4 mm	0	4		
5. Ulcer(s) on lips/nares	Non-bleeding	0	4		
	Bleeding	0	6		
6. Dyspnea with audible rales		0	2		
7. Open-mouth breathing		0	3		
8. Dehydration *		0	3		

The cumulative clinical score was calculated by identifying the highest daily score in each of the eight categories and calculating the sum of those values. A score of zero was assigned if a clinical sign was absent. * Dehydration was only counted in the cumulative clinical score if any of the other clinical signs were also present.

**Table 3:** Descriptive and enrollment data for shelter cats with naturally occurring upper respiratory tract disease enrolled in a randomized, placebo-controlled clinical trial of famciclovir.

Recorded Shelter Cat Parameters		Cats Included in Intention-to-Treat Analysis	
		Famciclovir Group (*n* = 11)	Placebo Group (*n* = 11)
Sex	Male	6	5
	Female	5	6
Location	Shelter A	6	7
	Shelter B	5	4
Estimated age in years	Median (range)	3 (0.46–14)	5 (0.5–12)
Duration of study enrollment in days	Median (range)	18 (2–21)	9 (4–21)
Clinical score on day of enrollment	Median (range)	4 (2–10)	3 (1–6)
Antibiotic administration during the study	Doxycycline alone	6	4
	Doxycycline + azithromycin	1	2
	Amoxicillin trihydrate/ clavulanate potassium	1	0

**Table 4:** RT-PCR detection results of upper respiratory tract disease pathogens for 21 shelter cats with naturally occurring upper respiratory tract disease enrolled in a randomized, placebo-controlled clinical trial of famciclovir.

Upper Respiratory Tract Pathogen	Famciclovir Group (*n* = 10) *	Placebo Group (*n* = 11)
Feline herpesvirus-1	5 ^†^	6 ^‡^
Feline calicivirus	4	5
*Chlamydophila felis*	2	2
*Bordetella bronchiseptica*	2	1
*Mycoplasma felis*	7	4

* One cat in the famciclovir group did not have samples submitted for RT-PCR; ^†^ Four/five cats had <38,000 viral particles/swab; ^‡^ Five/six cats had <38,000 viral particles/swab.
